# Empowering Cancer Survivors in Managing Their Own Health: A Paradoxical Dynamic Process of Taking and Letting Go of Control

**DOI:** 10.1177/10497323231158629

**Published:** 2023-02-24

**Authors:** Jonathan Avery, Roanne Thomas, Doris Howell, Claire-Jehanne Dubouloz Wilner

**Affiliations:** 1 School of Nursing, 8166 University of British Columbia, Vancouver, BC, Canada; 2 Department of Supportive Care, Princess Margaret Cancer Centre, University Health Network, Toronto, ON, Canada; 3 Department of Rehabilitation Sciences, 6363 University of Ottawa, Ottawa, ON, Canada

**Keywords:** cancer, grounded theory, patient empowerment, psychosocial, oncology, qualitative

## Abstract

In cancer care, gaps in support to help patients manage and live with the side-effects from cancer treatments have increased the emphasis on empowering patients to be more active and involved in managing their own health. However, empowerment in relation to promoting self-management behaviors is not well understood. Using the social constructivist grounded theory approach, our goal was to develop a theoretical understanding of this phenomenon in relation to the self-management behaviors of post-treatment cancer patients. Twenty-two post-treatment cancer patients participated in a semi-structured focused interview to co-construct with us how empowerment is defined, described, and experienced in relation to their capacity to self-manage. Through this co-construction, we defined empowerment as a process of personal growth, a display of fortitude and strength when participants confronted the impact of their illness that emerged in two dynamic and paradoxical ways: 1) establishing control over the impact of the illness as a means to maintain normalcy and to circumvent change over an eroding and changing sense of self and 2) relinquishing control over aspects of the illness deemed irrepressible and acknowledging and accepting change. When successful at establishing and/or relinquishing control, participants no longer viewed cancer as a threat, but re-interpreted their illness as also having a beneficial “empowering” experience and more capable of managing. Findings will guide the development of self-management interventions that use empowerment as a core construct.

## Introduction

In cancer care, the post-treatment survivorship phase of the cancer continuum is characterized by reduced access to professional and social support, thus requiring patients to become active and informed participants in managing their own care and learning how to address their own needs (Howell et al., 2019, 2021; McCorkle et al., 2011). “Cancer survivorship” can be defined in many ways (Berry et al., 2019; Lagergren et al., 2019; Ristovski-Slijepcevic, 2008, p. 5–7), but is most often understood as either the period following acute treatment (i.e., post-treatment) when the person has been disease free for a specific number of years or described more holistically as the period from the time of diagnosis, through the balance of life. While discussions remain about the most appropriate definition of cancer survivorship (Berry et al., 2019; Feuerstein, 2007), we define cancer survivorship as the period from the time of diagnosis through the balance of life. As a result of long-term persistent and often unpredictable physical and psychosocial sequelae from a diagnosis of cancer and its treatments, cancer is often described as an illness where survivors experience a loss of control and autonomy over their health and life (Vehling & Kissane, 2018). This loss of control and autonomy presents many challenges for survivors in adopting effective coping, behavioral and self-management strategies (Maunsell et al., 2014). Thus, there has been increasing interest in the concept of empowerment, its processes of re-establishing control and autonomy, and its clinical application in cancer survivorship care (Groen et al., 2015; Jerofke, 2013; Marzorati et al., 2018).

As a result of an increased emphasis on responsibility and active involvement, patient empowerment has emerged as an important indicator of efficient healthcare and successful symptom self-management in cancer care (Maunsell et al., 2014; McCorkle et al., 2011; Van Den Berg et al., 2013). Yet, empowerment as an experience is not well understood (Groen et al., 2015; Jørgensen et al., 2018). Research on empowerment is vast and encompasses theoretical, quantitative, qualitative, and mixed methods research; however, definitions are limited to anticipated outcomes, rather than defining empowerment according to the individuals’ perspectives of what empowerment means to them in relation to managing their own health (Aujoulat et al., 2007). Fewer studies explore empowerment from the perspective and experiences of cancer survivors (Jerofke, 2013; Jørgensen et al., 2018).

Broadly, empowerment is a process and outcome associated with establishing control and mastery over a situation (Rappaport, 1987). The term is considered a socially constructed concept with multiple meanings and pathways that vary depending on the individual and the context (Foster-Fishman et al., 1998). Within the cancer care context, the idea of an empowered patient comes from the dominant cancer survivorship discourse. Discourse in this context refers to a formal way of thinking that is influenced by the way ideas seen as relevant to the overarching concept (survivorship) are constructed and expressed (Conrad & Barker, 2010; Kaiser, 2008). The discourse in cancer survivorship presents a narrative of the illness experience that tells a story of a relentless fighter who had the strength to overcome the daunting aspects of the illness and its treatment. This person is viewed as someone who took control, capable of being involved, informed, and proactive in their care. As such, these are the characteristics that are implied in what it means to be diagnosed and treated and to survive cancer. This type of rhetoric dominates the media and cancer (psychosocial) research and cancer care governance (Bell, 2010; Ristovski-Slijepcevic & Bell, 2014; Sinding & Gray, 2005). Topics such as patient activation (van Maarschalkerweerd et al., 2017), patient engagement (French et al., 2015), and self-management (Howell et al., 2017) that place a strong emphasis on individual control, responsibility, and “empowerment” dominate the field (Sinding et al., 2011). Yet, there are several scholars who have challenged these deeply embedded assumptions (see Bell, 2014; Fox et al., 2005; Kaiser, 2008; Khan et al., 2012; Ristovski-Slijepcevic & Bell, 2014; Sinding & Gray, 2005). For example, Sinding et al. (2011) argue that the emphasis on patient empowerment as an essential element of quality cancer care rests on assumptions that people want to be in control and involved in their own health care when this may not be the case for everyone. Studies exploring patient participation preferences accentuate a range of differences in the desire for involvement along a spectrum from active to passive (Ernst et al., 2013; Lovell et al., 2014). Emphasizing patient participation on an individual who prefers not to be involved may contradict the deeply embedded assumptions from the cancer survivorship discourse.

Given the contradictions between the discourse and the individual illness experience, there are questions about the authenticity of the use of patient empowerment in cancer care. To understand the level of authenticity of patient empowerment in cancer care, the purpose of this study was to explore empowerment through a social constructivist lens and determine if, and if so how, empowerment is experienced in the specific context of cancer care from the perspective of those diagnosed and treated for cancer. The aim was to yield a more comprehensive understanding of a questionable phenomenon that is integral to the field of symptom self-management in cancer care. The following research questions (RQs) guided this exploration:

RQ1 In what ways do cancer survivors define, describe, and/or experience the processes of empowerment?

RQ2 What are the connections and relationships between processes of empowerment and self-management from the perspective of survivors?

By exploring these questions, we sought to define empowerment using the meanings, experiences, and perceptions of post-treatment cancer survivors to inform the role of empowerment in the post-treatment management of the disease.

## Methods

### Study Design

A qualitative research design drawing on the social constructivist grounded theory methodology informed by Charmaz (2014) was used. The choice to use the constructivist grounded theory was in our intent to uncover how (i.e., the processes by which) this phenomenon is experienced by a specific group of peoples (i.e., cancer survivors). Moreover, our choice was also based on Denzin and Lincoln’s premise that the researchers’ own beliefs and feelings about the world should be considered when choosing how to conduct research (Denzin & Lincoln, 2005). As researchers, we believe we are not passive objective observers, but rather active participants in the creation of meaning. Since these views are consistent with a social constructivist theoretical lens (Creswell, 2014; Mills et al., 2006), choosing constructivist grounded theory allowed us to become aware of and acknowledge our own worldviews and prior knowledge; this in turn allowed us to capture the experiences of the participants.

### Procedures and Participants

Semi-structured interviews with 22 post-treatment head/neck or breast cancer survivors who were in follow-up care at a tertiary cancer center located in Ontario, Canada, served as the primary means of data collection. Head/neck and breast cancer are two types of cancer that present different social characteristics and receive different levels of support, advocacy, and funding for research and program development (Binkley et al., 2012; Canadian Cancer Society’s Advisory Committee on Cancer Statistic, 2021; De Felice et al., 2015; Denieffe & Gooney, 2011; Howren et al., 2013; Molassiotis & Rogers, 2012). Since empowerment is an experience that is embedded in the social characteristics of an illness and the perceptions of the individual, there is a possibility that these differences in disease features and levels of support may cause variations in the meanings and processes of empowerment as perceived by those who are afflicted.

Interviews with head/neck or breast cancer survivors took place between November 2015 and September 2016. An interview guide was created to provide some structure for each interview (Table 1). Questions were kept broad and open-ended and included specific topics and subject areas to allow participants’ thoughts and perceptions about empowerment to emerge openly as they were asked to reflect on their illness experience. This was followed by directive questions about the emerging meanings and processes of empowerment. Since empowerment is a process and outcome associated with establishing control and mastery over a situation, we began each interview by asking participants about their life before they were diagnosed to prompt them to think about how their diagnosis might have disrupted their day-to-day life and how they attempted to re-establish themselves.

**Table 1.** Qualitative Interview Guide.

Topic	Question	Probes
Life before the diagnosis	“What was your life like before you were diagnosed with head and neck/breast cancer?”	• What did you do for work/leisure activities?
		• What were your main roles and relationships with your family/friends/community?
		• What was most important to you prior to your cancer diagnosis?
The experience of transitioning from treatment to post-treatment life	“Can you tell me about your experience being diagnosed and treated for head and neck/breast cancer?”	• What were some of the challenges?
		• How did you manage through these challenges?
		• What role did your family/friends/clinicians have in the management of these challenges?
		• How did your roles and responsibilities in your life change as you went through your treatment?
		• How did you try to manage these concerns? Can you expand on some of the strategies?
The experience of being diagnosed and treated for cancer	“What was the process like you when you finished your treatment?”	• What were or are some of the physical/psychological/social concerns you are/have faced since you have finished your treatments?
		• How did the treatment phase affect your relationships with your family/friends/co-workers? Can you speak more about this?
		• Can you talk about some of the support you received from family/friends/clinicians around the management of these concerns?
Empowerment	“Based on the experiences and challenges that you’ve expressed, what is your understanding of the word empowerment?”	• How important is this idea of control over your health and care?
		• What did you try to do to establish this level of control?
		• How does this level of control help you manage __________ (example of symptom)?
		• Can you walk me through the process?

Focusing on these different phases of the illness experiences allowed us to understand if, and if so how, empowerment emerged from the entire illness experience rather than focusing only on the post-treatment phase when professional and social support is significantly reduced. We wanted to understand from the patient’s perspective where in the illness experience the process of empowerment begins. Does the process of empowerment begin when a person is diagnosed with cancer, or when professional and social support is significantly reduced at post-treatment? No theoretical perspective or pre-conceived definition of empowerment was provided. This allowed the participants to define the concept from their perspective and past experiences. This decision was made to avoid the limitations presented in past research that explored pre-defined meanings of empowerment. This gave us the ability to explore empowerment from the perspective of the participants without super-imposing one specific understanding of empowerment over another. However, since constructivist grounded theory is an emergent design, we had flexibility to ask questions to explore any nascent themes concerning the experience (Charmaz & Belgrave, 2012). Therefore, probing questions often varied over the course of the data collection and analysis process. Interviews took place in person or over the phone. Interviews were conducted by the first author.

In addition to interviews, demographic information was collected via self-report from each participant before the interview to gain an understanding of the sample involved in the study to determine the heterogeneity of the sample and assess the transferability of the findings to other settings.

Purposeful sampling was used to identify post-treatment head/neck or breast cancer survivors who were interested in exploring their thoughts and feelings about becoming empowered in relation to managing the physical and psychosocial challenges they faced throughout their illness. The following inclusion criteria were used to identify participants: a diagnosis of head/neck or breast cancer; within 3 months to 5 years post-treatment; 18 years of age or older; able to describe their experience in English; and interested in exploring and sharing their experience of managing the physical and psychosocial challenges associated with diagnosis and treatment. Patients did not need to be diagnosed with both types of cancer to participate. Patients with recurrent or metastatic cancer; inability or refusal to provide informed consent; and any form of cognitive impairment (as judged by clinicians) and/or the inability to carry out an interview were excluded. Potential participants were approached in the head/neck and breast cancer outpatient clinics by either their oncologist or nurse to seek their interest in participating in the study. If interest was expressed, patients were provided further details about the study and consented to participate by the research coordinator. Our study was approved by the University Health Network (UHN) (CAPCR #15-8944) and the University of Ottawa (#H08-15-36) research ethic boards. Participants gave written and oral informed consent to participate.

### Data Analysis

Interviews were recorded and transcribed verbatim noting punctuation and pauses to help go beyond the words used by participants to describe the empowerment process. Interviews were analyzed by the first author using a three-level data coding process (initial, focus, and theoretical coding) outlined by Charmaz’s constructivist grounded theory method (Charmaz, 2014). For each interview, the analytic process began by reviewing each interview transcript as a whole and taking notes with initial thoughts to obtain an overall impression of the contents of each interview. Then, the interview transcript was reviewed line by line, and/or in segments (i.e., initial coding), to identify and highlight codes. Using the constant comparative approach, codes were then organized into themes and categories, relating them to previously analyzed data (i.e., focused coding). Relationships between data, codes, themes, and categories were mapped, and broader conceptual categories were formed to create the scheme that became the theoretical model of the empowerment process (i.e., theoretical coding). This analytical process occurred concurrently with data collection to facilitate theoretical sampling. Theoretical sampling was a strategy we used later in the theory building process to use previously collected data to determine what themes needed to be explored further to gain a deeper analytical understanding of empowerment (Draucker et al., 2007). For example, control was a theme that participants discussed during early interviews. If a participant did not organically discuss control during a later interview, we asked directive probes to have them elaborate on whether control was a key characteristic of their views of empowerment. This concurrent process is consistent with the grounded theory method (Draucker et al., 2007; Walker & Myrick, 2006). Co-authors participated in the data analysis process by providing feedback on the progression of the coding when the primary author presented on the emerging results. Rigor was maintained by staying as close as possible to the words used by participants when analyzing and coding data and by checking the theoretical construction generated against participants’ meanings of the phenomenon during interviews. In addition, reflexive journaling, writing memos, and keeping field notes were used to critically examine the first author’s influence on the research process, and on the co-creation of meaning with research participants, to determine the most relevant themes that represent the process of empowerment. These procedures were based on Lincoln and Guba’s (1985) criteria for producing qualitative research of high quality and ensure that participants’ views were fully captured while the researcher’s thoughts and biases were not superimposed (Gentles et al., 2014). While engaging in these procedures, we paid specific attention to differences in the ways empowerment was articulated between head/neck and breast cancer survivors. The qualitative software program ATLAS.ti aided with the organization and coding of the interview transcripts.

## Results

### Participant Characteristics

Our final sample (*N* = 22) included 13 individuals with head/neck cancer (10 men and 3 women), 8 women with breast cancer, and 1 woman who had a diagnosis of breast cancer followed 10 years later by a diagnosis of head/neck cancer. A heterogeneous sample across age, gender, marital and employment status, annual net income, and treatment type, as well as the length of time from treatment to post-treatment recovery, was recruited (Table 2).

**Table 2.** Participant Characteristics (*N* = 22).

Participant Characteristic	Frequency (%)	Mean	SD	Range
Age (years)		54	13.2	26–68
Sex				
Male	10 (45.0%)			
Female	12 (55.0%)			
Cancer type				
Head/neck	14 (63.6%)			
Breast	9 (40.9%)			
Education				
High school or less	6 (27.3%)			
College/university education or above	16 (72.7%)			
Employment status				
Employed	10 (45.5%)			
Retired	5 (22.7%)			
Unemployed/on disability	6 (27.3%)			
Student	1 (4.5%)			
Country of birth				
Canada	16 (72.7%)			
Other	6 (28.6%)			
Current relationship status				
Married/life partner	15 (68.1%)			
Divorce/separated	2 (9.1%)			
Never married	4 (18.2%)			
Widowed	1 (4.5%)			
Annual net income				
Over $90,000	6 (27.3%)			
$61,000–$90,000	7 (31.8%)			
$30,000–$60,999	5 (22.7%)			
Below $30,000	3 (13.6%)			
Prefer not to answer	1 (4.5%)			

### Findings

**Figure 1.**
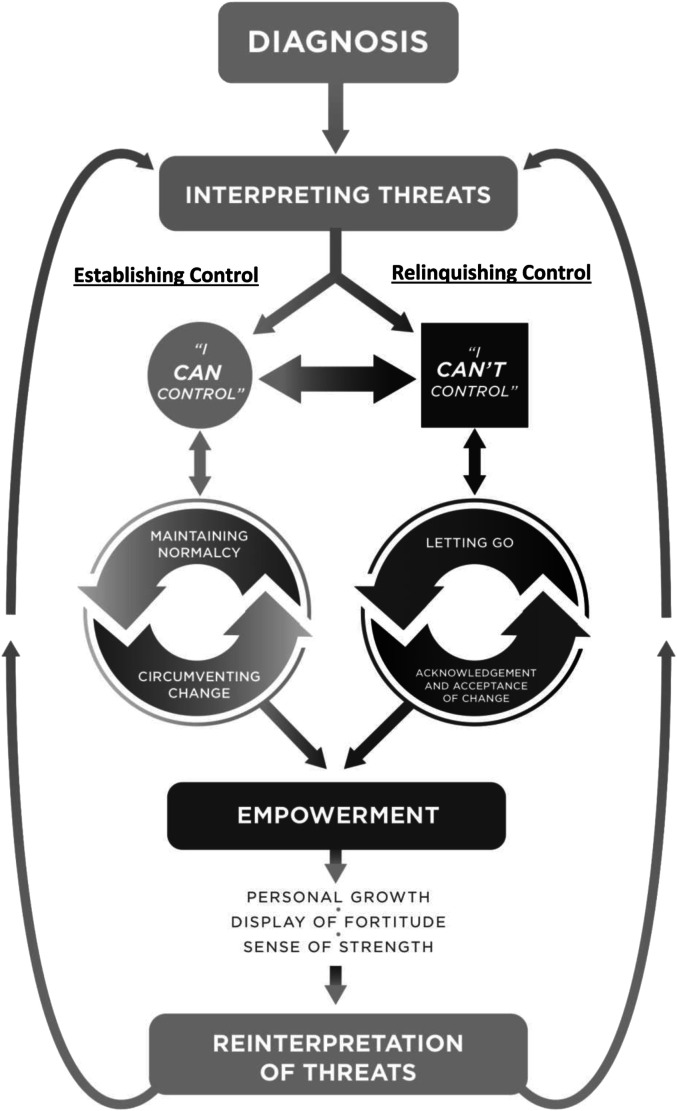
The model of empowerment.

Our findings illustrated that participants defined empowerment as a process of personal growth and a display of fortitude and strength when confronting the impact of their illness and emerged in two concurrent, dynamic, and paradoxical ways: 1) establishing control over the impact of the illness as a means to maintain normalcy and to circumvent change over an eroding and changing sense of self and 2) relinquishing control over aspects of the illness deemed irrepressible and acknowledging and accepting change. Establishing and/or relinquishing control involved a number of different actions that participants took to manage the consequences of their illness. We group these actions as sub-themes. To establish control, participants attempted to i) control how and in which ways they disclosed their illness to others; ii) how they incorporated their illness into daily life; and iii) how they advocated for their needs. Participants attempted to relinquish control by i) “letting go” of the idea of control and ii) accepting/acknowledging that there were circumstances associated with their illness that could not be controlled. When successful at establishing and/or relinquishing control, participants no longer viewed cancer as just a threat (initial interpretation), but re-interpreted their illness as also having an empowering experience. Interpreting and re-interpreting threats emerged as the core category of empowerment process (Figure 1) (Table 3).

**Table 3.** Themes and Participant Quotations.

Theme	Sub-Theme	Exemplification Quotes
1. Establishing control	Controlling levels of disclosure	• “…they took out a portion of my tongue and a portion of my saliva gland. They basically cut across my throat and now I am left with a ‘turkey neck’… and I’ve learned to deal with it. Everybody bought me scarfs. I have so many scarfs… it was just so horrible looking… and you couldn’t use makeup on it or anything like that…” (Lizzy, 68-year-old head/neck cancer survivor)
		• “Telling my kids was horrible. I told my parents first and that was hard. Because as a parent you don’t ever want to see your kids sick. And I didn’t tell anybody and I wouldn’t let my husband tell anybody until I’d absolutely knew what I was dealing with, because I didn’t want people to worry.” (Sarah, 49-year-old breast cancer survivor)
	Incorporating illness into daily life	• “… One of the key concerns was my woodworking, that before surgery I could wear a regular gauze mask… Well, there’s no method of filtering the dust for my situation now… I’ve tried to modify a regular type of mask to fit over the stoma…” (Jason, 61-year-old head/neck survivor)
		• “I still carry it [the device]… it’s pretty handy… where you can write on it and you can rub it off… It’s not a whiteboard… You just touch, there’s a little button at the top, and whatever you write just disappears… I was determined to learn how to talk because I like to talk” (Billy, 65-year-old head/neck survivor)
	Advocating for one’s needs	• “… getting to your point about empowerment, I felt helpless and useless and I was going to myself ‘Am I really going to have to wait a month and a half just to get a CAT scan for this thing… I called back and I said this is ridiculous. I can’t wait a month and a half for something that is really serious… Sure enough two days later I went in for a scan…” (Josh, 57-year-old head/neck cancer survivor)
		• “…So I needed to talk to another hospital… the pain management area… If this stuff does a little bit better for me… so I asked Dr. X about oil from marijuana. I am curious about that…” (Frank, 53-year-old head/neck survivor)
2. Relinquishing control	Letting go	• “…Well I do have people that stare at me. But I don’t really care. If they don’t like me they can stare the other way…” (Betty, 55-year-old head/neck cancer survivor)
		• “I think life just has to carry on. You just gotta get back into it… life just continues, and you have to keep going with it…” (Erin, 48-year-old breast cancer survivor)
	Acknowledgment and acceptance of change	• “…when I started socializing again…I was exuberant because I went through hell and everything is over now so ‘look at my feeding tube! Look this is my optimator!…’ My wife would hold me back. ‘Don’t be so exuberant with sharing your experiences with everybody’. But, I don’t mind talking about it at all or telling people. It’s kind of like I am revitalized.” (Josh, 57-year-old head/neck survivor)
		• “… it [cancer] made me more aware… Because all my buddies know, ‘you never smoked’. I said, ‘see it can get anybody’. It happens, that’s all… and you can’t sit back on something like this. You’ve just got to recognize what you’ve got… It’s the new normal.” (Billy, 65-year-old head/neck cancer survivor)
3. The emergence of empowerment: Successfully taking and letting go of control	Interpreting and re-interpreting threats	• “I think having [cancer] made me realize that you don’t have much control in life… I would not say that everyone should have cancer and to go through it… but I think it was beneficial. For me personally, it changed my perspective on a lot of stuff.” (Jason, 51-year-old head/neck cancer survivor)
		• “It [cancer] empowered me to talk to people about this. It’s a little better life actually. It gives you a totally different perspective on life…” (Sharon, 67-year-old head/neck survivor)
		• “You don’t see life the same way after cancer. I am not sure but I think I can manage conflicts or challenges better. I don’t worry much about things. If I face a challenge I know I will come out with a solution.” (Maria, 37-year-old breast cancer survivor)

#### Establishing Control

When participants were diagnosed and treated for cancer, they felt a loss of control and autonomy over their life. To cope, participants attempted to re-establish this control in order to maintain some form of normalcy and to circumvent change over an eroding and changing sense of self. Participants used different strategies to re-establish this control. They did so by controlling levels of disclosure, incorporating symptom management into daily life, and advocating for one’s needs.

##### Controlling Levels of Disclosure

Precaution was taken by many participants when disclosing aspects of their illness to other people including family members, friends, and employers/colleagues because of various fears associated with worrying others and concerns about being treated differently. In this way, participants tried to maintain control by deciding whether and how to disclose. For example, Jason (51-year-old head/neck cancer survivor) purposely isolated himself from his colleagues as to avoid unwanted reactions or attention. He noted: “*… when I went [back to] to work, I really just stayed in my office because I could not speak properly and then everyone would go* ‘*What’s wrong?*’ *But, I did not want to make a big deal of it….*”

In self-isolating, Jason was able to control levels of disclosure to avoid people treating or viewing him differently, thus circumventing changes to an eroding and changing sense of self related to his inability to speak properly.

##### Incorporating Illness into Daily Life

In many circumstances, symptoms or treatment side-effects could not always be concealed. Thus, rather than controlling levels of disclosure, participants learned to incorporate aspects of their illness into daily living as a way to feel in control. Stephanie (26-year-old breast cancer survivor) incorporated symptoms into daily living by choosing to have reconstructive surgery after having a double mastectomy to preserve her identity as a young woman. Stephanie noted: “*…I felt kind of silly to go through this horrible pain just so that I would have breasts. But I know in a year from now I will be happy that I did…I’m really young and I didn’t ever want to get rid of my breasts….*” By incorporating this aspect of her disease into daily life (choosing to reconstructive surgery), Stephanie was able to circumvent an eroding and changing sense of self by choosing to have reconstructive surgery.

##### Advocating for One’s Needs

Advocating for one’s needs occurred when participants thought that they were not receiving immediate or appropriate care or were having difficulty managing symptoms. These circumstances triggered feelings of distress and a sense of helplessness that their illness would worsen and would have a greater impact on their daily life if concerns were not raised and action not taken. Sharon (67-year-old head/neck and breast cancer survivor) was having a difficult time managing the toxicities of her chemotherapy and asked her medical oncologist to reduce the strength of her treatment. Advocating for this need was monumental in providing this participant with a sense of control and enabled her to perceive that she was capable of reducing the impact of her treatment so that she would feel more normal: “*… I was going to maintain part of my normal routine. That was very important to me… Fortunately they reduced the chemo… I said to myself ‘I am never vomiting again with this’… and I never did!*”

#### Relinquishing Control

As participants attempted to re-establish control, many came to the realization that there were aspects of their illness that could not be controlled. When this realization occurred, participants tried to “let go” and acknowledge and accept that change was inevitable and should be embraced, rather than controlled and/or avoided (Table 3).

##### Letting Go

Letting go occurred when participants learned what aspects of their illness could not be controlled—when they came to the realization that controlling certain aspects of their illness was either too difficult and/or not possible. Paul (65-year-old head and neck survivor) suffered nerve damage from removal of cancerous lump from the inside of cheek and now lives with an alerted appearance. He came to the realization that he could not control or hide alerted appearance prompting him to let go, saying *say* “*… I can’t let this [his altered appearance] to control me… So I said the hell with it. I put on a pair of sun glasses and go! And that’s what I did*.” This realization prompted participants to relinquish efforts of trying to control circumstances that they deemed too difficult and/or not possible. Thus, rather than try to establish control to circumvent change, participants attempted to let go of efforts to manipulate these situations. For example, Jackie (46-year-old breast cancer survivor) said: “*...there are certain things you don’t have control over… So I’ve learned that I’m only going to control what I can, and the other stuff I just have to let it go.*” For Jackie, “letting go” included her acknowledgment that preventing cancer was beyond her control: “*I did everything I thought I could do to not get cancer and I still got it. I realized you can’t control it.*” This ability to let go provided Jackie with a sense of freedom that she could move forward with her recovery, noting: “*…I feel like it’s a huge burden that’s been lifted. I feel a lot freer that I’m on my journey to recover*.”

##### Acknowledgment and Acceptance of Change

Along with letting go, participants expressed their need to acknowledge and accept that there were circumstances associated with their illness that could not be controlled. However, for many participants, this type of acknowledgment and acceptance was fraught with challenges. Tatianna (35-year-old head/neck cancer survivor) was struggling with the notion of not having control: “*… this situation [cancer] has been very hard because it showed me that I can’t control anything. I can make plans but then one day ‘boom!’ and it’s all going to change. And now I am trying to take one day at a time…*.” Her decision to try to live life one day at a time is not only an illustration of her attempt to let go but is also an example of how she is attempting to accept and acknowledge her new reality of the limited control she perceived over her life. For participants who were successful at acknowledging and accepting of change, they saw themselves as more capable of letting go and of embracing the afflictions of their illness.

### The Emergence of Empowerment: Successfully Taking and Letting Go of Control

Empowerment emerged as participants moved between controlling the impact of the illness as a means to circumvent change, and by letting go and accepting and acknowledging aspects of the illness that could not be controlled. When successful, participants no longer viewed cancer as only a threat, but re-interpreted the illness as a beneficial experience associated with personal growth and an opportunity to display a sense of strength and fortitude associated with surviving cancer. This re-interpretation of the illness experience was the epitome of the empowerment experience. Interpreting and re-interpreting threats associated with cancer emerged as the core process that binds these paradoxical processes of empowerment (i.e., taking and letting go of control) together.

#### Interpreting and Re-Interpreting Threats

For participants, a diagnosis of cancer and its treatments were seen as threats that acted as the catalyst to begin the process of empowerment. Cancer was perceived as an intrusive threat to one’s sense of self and livelihood leading to suffering and life uncertainty. For Gerard (61-year-old head/neck survivor), this meant the risk of losing his ability to fully participate in his post-retirement passion of carpentry. “*… One of the key concerns was my woodworking, that before surgery [laryngectomy] I could wear a regular gauze mask… Well, there’s no method of filtering the dust for my situation now….*” This threat propelled Gerard to attempt to establish control by trying to find ways to incorporate the impact of his illness into day-to-day life so he could continue to participate. “*I’ve tried to modify a regular type of mask to fit over the stoma, but that didn’t work… I didn’t expect the mask to be so difficult….*” He has yet to be successful. Even so, he still feels empowered by his efforts. When asked to describe if any aspects of his experience with cancer proved to be a source of empowerment, Gerrard said the following:

… *The fact that I was able to work out again… able to lift more weights than I was pre-surgery… and the fact that I can still go out and play golf and still blow everybody away! My lifestyle has not really changed except for my ability to talk*…

Gerard’s ability to establish a certain level of control over the impact of his illness to circumvent any significant lifestyle changes has proven to be a source of pride and psychological strength that he associates with being empowered. In addition, Gerard also acknowledged certain consequences of his illness that cannot be controlled. When referring to his difficulties speaking, Gerard noted the following: “*There is no fix for this. This is the way you’re going to be the rest of your life and my response of ‘ok’….*” This is an acknowledgment that also seemed to have contributed to Gerard’s emerging sense of empowerment:

…*six months ago, seven months ago, I’m not the same person as I am today. That person would not have been able to deal with the surgery and the ultimate loss. I mean mentally, I didn’t feel I was strong enough for that… I don’t know why, but I’ve never had as positive an attitude about things. But I do now*…

This example demonstrates that Gerard’s emerging sense of empowerment seemed to emerge from two distinct but simultaneous paradoxical processes. The first process is associated with Gerard’s ability to establish control over the impact of the illness as a means to circumvent an eroding and changing sense of self observed by his ability to remain engaged in activities that brought him meaning before his diagnosis. At the same time, Gerard also relinquishes control over aspects of the illness he deemed irrepressible (his ability to talk) and incorporates this aspect into a new and “empowered” identity, reflected in his newfound strength and positive attitude. Based on Gerrard’s experience, it appears that cancer is no longer viewed as simply a threat, but now viewed/re-interpreted as a beneficial experience.

This type of illness re-interpretation occurred for other participants. Sara, 49-year-old breast cancer survivor, went through similar processes of interpreting and re-interpreting the threats associated with her diagnosis. Sara was at first apprehensive and refrained from disclosing her illness to her employees out of fear she would appear weak and worry others. When she decided to forgo this type of control, she stated the following: “*…I was really relieved that I told them and I could just you know, be a bit more myself and let down my guard*.” She later explained that these types of experiences gave her a sense of empowerment, saying: “*…These experiences have empowered me. It’s made me much more confident in who I am…*.” Thus, Sara’s experience reflects these processes of moving between taking control (refraining from disclosing her illness) and then “letting go” (eventually disclosing her illness), which brought her a newfound confidence in her changing sense of self. In addition, Sara gained an appreciation for going through the illness:

*So I think [cancer] forced me to be a better person. I have more patience and more time to give to my family and I am thankful of that… All the bad stuff has given me some really good things… It was a bad experience. It was a year of my life but, I have really great things to take away from it… so it [cancer] has changed me for sure. I’m glad I have cancer… And I feel very proud of you know, the fight*…

This re-interpretation of the illness did not occur instantaneously, but as a cumulative process of moving between taking and letting go of control. For Sara, moving between these processes opened up to new ways of understanding and incorporating the illness into daily living. This was the epitome of these paradoxical processes of empowerment—of taking and letting go of control.

## Discussion

Our findings illuminate how empowerment is understood and experienced by those living 3 months to 5 years after their treatment for cancer. Prior research has shown that definitions of empowerment are limited to an anticipated outcome, such as establishing control over and capable of managing one’s own health, rather than defining empowerment according to the individuals’ perspectives of what empowerment means to them (Aujoulat et al., 2007; Jerofke, 2013). Participants from our study described empowerment as a dual social process of establishing control over the impact of the illness and relinquishing control over aspects of the illness deemed irrepressible. When these findings are compared and contrasted with traditional ways of understanding empowerment, a number of insights can be drawn, and recommendations made that speak to the applicability of this theory of empowerment to cancer care.

First, most studies in cancer care approach empowerment as equivalent to control, with fewer inquiries exploring the dynamic ways this phenomenon can emerge. For example, in their phenomenological inquiry of empowerment from the perspective of 12 hematology cancer patients, Bulsara et al. (2004) illustrate that accepting periods of no control is an integral characteristic of the process of empowerment and an antecedent to re-establishing control. However, acceptance of limited control was understood as a temporary period of disempowerment rather than a permanent fixture of a new and empowering reality that participants of our study emphasized. Similarly, in a concept analysis of empowerment in cancer survivorship, Jerofke (2013) postulates that empowerment is a dynamic process of “navigating periods of being ‘well’ and periods of being ‘ill’ associated with alternating periods of feeling empowered [in control] and disempowered [not in control]” (p. 160). Here, a lack of control is understood as a disempowering characteristic. For participants of our study, accepting periods of no control was associated with developing a new and empowered sense of self, rather than a sense of disempowerment. This way of experiencing empowerment is more akin to theories of post-traumatic growth wherein people who have suffered highly adverse, traumatic events derive benefit from cultivating a stronger sense of self (Occhipinti et al., 2015; Shand et al., 2015). Yet, there are notable differences between post-traumatic growth and our results. In a qualitative grounded theory study exploring the processes of post-traumatic growth in a sample of 24 Chinese breast cancer survivors, Zhai et al. (2021) found that the intentional pursuit of personal growth when faced with the trauma from cancer was a core element of the experience and grounded in a process of enhancing or letting go of control. Similar results were reported by Morteza et al. (2017) wherein they concluded that the central concept of post-traumatic growth is the intentional pursuit for self-actualization to cope with the trauma inflicted from a diagnosis of cancer. Our theory of empowerment is different in that it was not an all or nothing, binary or fixed experience to achieve a specific outcome of self-actualization, but rather more nuanced and evolving assessment over the ongoing challenges associated with being diagnosed and treated for cancer. The survivors’ recognition and acknowledgment that some aspect of treatment, symptom experiences, and re-integration into work and social life was beyond their control, provided some with confidence in their judgment and ability to come to terms with their circumstance and more capable to managing their own care.

Empowerment is considered a multi-level construct that begins at the individual level (Schulz et al., 1995). At the individual level, empowerment is commonly approached by developing a person’s knowledge, competency, and sense of self-efficacy in addition to building the determination to address any threat or sense of helplessness when a sense of control or autonomy is lost. Iterations of Bandura’s theory of self-efficacy and Deci and Ryan’s theory of self-determination form the basis of this understanding (see Aujoulat et al., 2007; Cattaneo & Chapman, 2010). Yet, these theories do not seem to consider how empowerment might also occur in situations perceived as beyond the control of the individual.

Participants in our study noted several hardships that were beyond their control. Physical impairments associated with eating and speaking as well as facial changes were commonly reported by those participants with head/neck cancer as aspects of their new reality that could not be changed. Those with breast cancer noted other types of difficulties related to the physical and psychosocial trauma associated with more complex cancer treatments; specifically, brain fog, chronic pain and fatigue, premature menopause, lymphedema, and the distress associated with full or partial mastectomies were common struggles. When a hardship was interpreted as beyond the control of the participants, they engaged in the process of “letting go” by acknowledging and accepting of change to incorporate aspects of the illness deemed irrepressible into a new and empowered identity. These strategies reflect the dynamic process of empowerment of moving between establishing and relinquishing control as participants learned what aspects of their illness were manageable in order to maintain some form of normalcy, while also adapting or adjusting to their new reality. This dual process of empowerment illustrated how participants learned to effectively manage the consequences of being diagnosed and treated for cancer.

What may be emerging from this grounded theory are some of the long-term implications of constant fluctuations between feeling in and out of control. Cancer is an illness that can have a life-long impact, which suggests constant fluctuations between periods of feeling well (in control) and feeling sick (not in control). Results suggest that a possible long-term implication is eventually surrendering to the idea of not being in control over certain aspects of the illness, which for participants was a form of liberation. The notion of being liberated provided a sense of power over one’s own health, which appeared to enhance the well-being and capacity for participants to manage the hardships of their illness that they perceived to be beyond their control. However, this view of empowerment is counter-intuitive to traditional ways this phenomenon is understood. This view also contradicts the dominant cancer survivorship discourse that suggests that stepping up and taking control will empower the individual to successfully manage and survive the illness. Instead, empowerment may be a single process that occurs at the intersection of controlling and letting go; learning to discern what is within the patient’s power, what is not, and when to exercise it.

This study has some notable limitations. This model of empowerment is a co-creation between the researcher and post-treatment survivors of head/neck or breast cancer with a mean of 54 years of age. The processes of empowerment could very well be different for those with a different diagnosis and at other phases in the cancer continuum including end of life. Would the processes be the same for adolescent and young adults diagnosed with an advanced stage cancer? In addition, our participants do not reflect Canadian diversity regarding gender, race, and socio-economic status. Most of our participants are college/university educated, are married or in a life partnership, and defined themselves as men or women. Given that empowerment is a socially constructed concept with multiple meanings and pathways that vary depending on the individual and the context (Foster-Fishman et al., 1998), its processes would have to be explored within different socio-economic and cultural sub-groups to determine the transferability of this model of empowerment to other cancer populations. In addition, this grounded theory of empowerment is a reflection of the co-construction of meaning between the first author and the participants of this study. This limitation reflects the theoretical lens (social constructivism) used to explore this phenomenon. Within this lens, there is possibility that the interpretation of the data shaped the findings of this study and that the experiences of the research participants are not accurately represented. However, the rigorous procedures used when collecting and analyzing the data should ensure that the findings accurately represent the experiences of those interviewed. Reflexive journaling, writing memos, and taking field notes were used to ensure emerging insights were based on the experiences of the participants and not solely a projection of the first author’s thoughts and feelings. As an example, field notes were very helpful because it allowed the first author to note on how the interview setting, his body language, and how he spoke to each participant could be adjusted to ensure he understood and interpreted what each participant disclosed. It was found that repeating and/or paraphrasing and summarizing participants’ responses after each question was a useful technique to confirm the understanding and interpretation of the participants’ thoughts and feelings. Repeating and/or paraphrasing was also useful in building rapport with each participant by helping them feel heard and understood.

## Implications for Practice and Future Research

Despite growing acceptance of the concept of patient empowerment and its importance for improving patient engagement in health care and health outcomes (Chen et al., 2016; Zimmerman, 1995), uncertainty still exists about how to facilitate and support empowerment in cancer care. Interventions that use empowerment as a core concept in cancer care have viewed this concept as a process of gaining greater control over the decisions and actions affecting their health. Few have incorporated the view that empowerment may be a single process that begins every time an individual perceives their illness as a threat to their sense of self, which propels them to determine which aspects of cancer survivorship are controllable and which are not—that the actual sense of empowerment may lie at the intersection of controlling and letting go; learning to discern what is within the patient’s power, what is not, and when to exercise it. The cancer survivors’ choice to act or not and learning to discern what is within their power can be facilitated by focusing the better communication and partnership between the survivor and their providers. Future research could explore how to facilitate this conversation every time they perceive a threat to their sense of self at any point during treatment, post-treatment, and long-term follow-up. Care models that promote patient involvement in care, shared decision-making, support planning, and self-management support at each of these points are being tested in the United Kingdom that perhaps could be a model for cancer organizations, globally (NHS, 2015).
